# Adsorption of gas molecules on monolayer MoS_2_ and effect of applied electric field

**DOI:** 10.1186/1556-276X-8-425

**Published:** 2013-10-17

**Authors:** Qu Yue, Zhengzheng Shao, Shengli Chang, Jingbo Li

**Affiliations:** 1 College of Science, National University of Defense Technology, Changsha 410073, China; 2 State Key Laboratory for Superlattice and Microstructures, Institute of Semiconductors, Chinese Academy of Sciences, Beijing 100083, China

**Keywords:** Monolayer MoS_2_, Molecular adsorption, Electric field, First-principles calculations, 68.43.-h, 73.20.Hb, 73.22.-f

## Abstract

Using first-principles calculations, we investigate the adsorption of various gas molecules (H_2_, O_2_, H_2_O, NH_3_, NO, NO_2_, and CO) on monolayer MoS_2_. The most stable adsorption configuration, adsorption energy, and charge transfer are obtained. It is shown that all the molecules are weakly adsorbed on the monolayer MoS_2_ surface and act as charge acceptors for the monolayer, except NH_3_ which is found to be a charge donor. Furthermore, we show that charge transfer between the adsorbed molecule and MoS_2_ can be significantly modulated by a perpendicular electric field. Our theoretical results are consistent with the recent experiments and suggest MoS_2_ as a potential material for gas sensing application.

## Background

Sensing gas molecules, especially toxic gas, is critical in environmental pollution monitoring and agricultural and medical applications [1]. For this reason, sensitive solid-state sensors with low noise and low power consumption are highly demanded. While sensors made from semiconducting metal oxide nanowires [2,3], carbon nanotubes [4,5], etc. have been widely studied for gas detection for some time, graphene as a novel sensing material has further stimulated strong interests in the research community since Schedin et al. [6] demonstrated that a micrometer-sized graphene transistor can be used to detect the ultimate concentration of molecules at room temperature, presenting a pronounced sensitivity many orders of magnitude higher than that of earlier sensors. The graphene-based sensor is actualized by monitoring the change in resistivity due to the adsorption or desorption of molecules, which act as charge acceptors or donors [7-9]. It is shown that sensitivity of this sensor can be further improved through introduction of the dopant or defect in graphene [10-13]. Despite these achievements, researchers continue to seek for novel sensitive sensors similar to or even more fascinating than graphene gas sensors.

Recently, two-dimensional monolayer MoS_2_, a kind of transition metal dichalcogenide, has attracted increasing attention because of its versatile and tunable properties for application in transistor, flexible optoelectronic device, photodetector, and so on [14-19]. Unlike graphene which lacks a band gap and needs to be engineered to open the gap for practical application, pristine monolayer MoS_2_ has a direct band gap of 1.9 eV [20] and can be readily used to fabricate an interband tunnel field-effect transistor (FET) [21-26]. In this context, Radisavljevic and co-workers [21] first reported a top-gated FET on the basis of monolayer MoS_2_, which possesses a room-temperature current on/off ratio exceeding 10^8^ and mobility of 200 cm^2^ V^-1^ s^-1^. At the same time, the success of graphene-FET sensors also greatly inspires the intensive exploration of MoS_2_ as a sensing material. Since monolayer MoS_2_ holds a high surface-to-volume ratio comparable to graphene, a MoS_2_-based gas sensor is expected to have excellent sensing performance as well. More recently, FET sensors made from mechanically cleaved monolayer and multilayer MoS_2_ have been demonstrated, which exhibit high sensitivity for NO gas with a detection limit down to 0.8 ppm [27]. The superior sensitivity for NO_2_ has been observed in a flexible FET sensor array on a polyethylene terephthalate (PET) substrate based on a MoS_2_ channel and reduced graphene oxide (rGO) electrodes [28]. Compared to the rGO-FET sensor, this novel sensor array displays much higher sensitivity, which can even be enhanced by up to three times via functionalization of MoS_2_ with Pt nanoparticles.

Although the MoS_2_-FET sensor for nitride oxide has been experimentally realized, the underlying mechanisms regarding how NO_*x*_ molecules interact with the MoS_2_ surface and affect the electronic properties are not clear. Moreover, the response of MoS_2_ upon exposure to other gas molecules like H_2_, O_2_, H_2_O, NH_3_, CO, etc. remains to be examined either. In order to fully exploit the possibilities of a MoS_2_-based gas sensor, a systematic study on the adsorption of gas molecules on a MoS_2_ surface is thus desired from a theoretical point of view. In this work, using first-principles calculations, we first determine the most stable configuration for gas molecules adsorbed on monolayer MoS_2_, as well as the corresponding charge transfer between them. Modification of the electronic properties of host monolayer MoS_2_ due to the molecule adsorption is then examined. Furthermore, the effect of an external electric field on the charge transfer is also discussed. To the best of our knowledge, no prior theoretical work has been conducted on these issues.

## Methods

First-principles calculations are performed using the Vienna *ab initio* simulation package (VASP) [29,30] on the basis of density functional theory (DFT). The exchange-correlation interaction is treated by local spin density approximation (LSDA). Spin-polarized calculations are also carried out with generalized gradient approximation (GGA) in some specific cases. A cutoff energy of 400 eV for the plane-wave basis set and a Monkhorst-Pack mesh [31] of 5 × 5 × 1 for the Brillouin zone integration are employed. In order to eliminate the interaction between two adjacent monolayer MoS_2_, a vacuum layer larger than 15 Å is adopted in the calculations. All the structures are fully relaxed by using the conjugate gradient method until the maximum Hellmann-Feynman forces acting on each atom is less than 0.02 eV/Å. By means of Bader analysis [32], charge transfer between the monolayer substrate and the adsorbate is obtained. The electric field in VASP is actualized by adding an artificial dipole sheet at the center of the simulation cell.

## Results and discussion

We consider the absorption of H_2_, O_2_, H_2_O, NH_3_, NO, NO_2_, and CO on two-dimensional monolayer MoS_2_. A 4 × 4 supercell of monolayer MoS_2_, with a single gas molecule adsorbed to it, is chosen as the computational model. The optimized lattice constant of monolayer MoS_2_ is 3.12 Å, and consequently, the distance between two neighboring gas molecules is larger than 12 Å. The monolayer MoS_2_ consists of a monatomic Mo-layer between two monatomic S-layers like a sandwich structure, in which Mo and S atoms are alternately located at the corners of a hexagon. In order to determine the favorable adsorption configuration, four adsorption sites are considered, namely, H site (on top of a hexagon), T_M_ (on top of a Mo atom), T_S_ (on top of a S atom), and B site (on top of a Mo-S bond). The gas molecule is initially placed with its center of mass exactly located at these sites. For each site, configurations with different molecular orientations are then examined. Take NO as an example, three initial molecular orientations are involved, one with NO axis parallel to the monolayer and two with NO axis perpendicular to it, with O atom above N atom and O atom below N atom [see Additional file 1 for more detailed adsorption configurations]. The adsorption energy is calculated as $E_{a}=E_{{\text{MoS}}_{2}+\text{molecule}}-(E_{{\text{MoS}}_{2}}+E_{\text{molecule}})$, where $E_{{\text{MoS}}_{2}+\text{molecule}}$ is the total energy of MoS_2_ with an absorbed molecule and $E_{{\text{MoS}}_{2}}$ and *E*_molecule_ are the total energies of pristine MoS_2_ and isolated molecule, respectively. A negative value of *E*_*a*_ indicates that the adsorption is exothermic.

**Figure 1:**
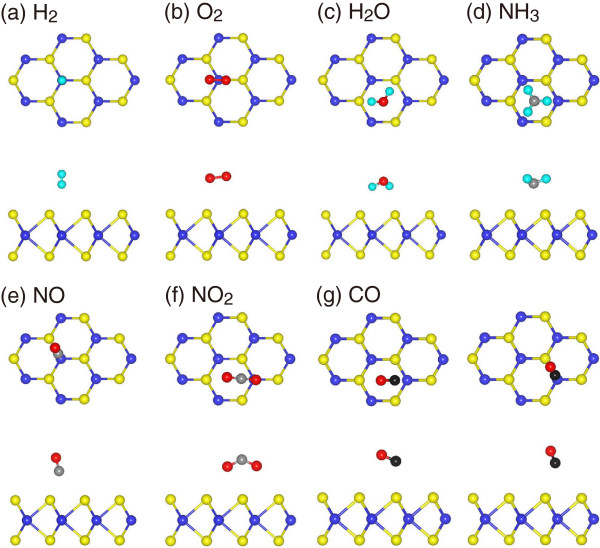
**Adsorption configurations.** Top and side views of the most favorable configurations for **(a)** H_2_, **(b)** O_2_, **(c)** H_2_O, **(d)** NH_3_, **(e)** NO, **(f)** NO_2_, and **(g)** CO on monolayer MoS_2_. The blue and yellow balls represent Mo and S atoms, whereas the cyanine, red, gray, and black balls represent H, O, N, and C atoms, respectively.

Table 1 summarizes the calculated values of equilibrium height, adsorption energy, and charge transfer for the adsorption of gas molecules on monolayer MoS_2_. The values for each adsorbate correspond to its favorable adsorption configurations obtained at different sites. The equilibrium height is defined as the vertical distance between the center of mass of the molecule and the top S-layer of the MoS_2_ sheet. Note that the adsorption energies are often overestimated at the LDA level, but this is not very essential here because we are primarily interested in the relative values of adsorption energies for different configurations and finding the most favorable one among them. From Table 1, we see that for both H_2_ and O_2_, the T_M_ site is found to be their most favorable site with the adsorption energies of -82 and -116 meV, respectively. The corresponding structures are shown in Figure 1a,b. Nevertheless, it seems that the two molecules adopt distinct orientations. While H_2_ has an axis perpendicular to the monolayer, that of O_2_ is nearly parallel to the monolayer with its center of mass on top of the T_M_. H_2_O, NH_3_, and NO_2_ are preferably adsorbed at the H site, resulting in the adsorption energies of -234, -250, and -276 meV, respectively. Structures for the three systems are shown in Figure 1c,d,f. Contrary to the configuration for H_2_O where H-O bonds adopt tilted orientation with H atoms pointing at the monolayer, all the H atoms of NH_3_ point away from the monolayer. NO_2_ is bonded with O atoms close to MoS_2_. In our calculations, H_2_, O_2_, H_2_O, and NH_3_ fail to have stable configuration at the B site; this is because they tend to migrate to other sites during structural relaxations. In contrast, the configuration with the center of mass located at the B site is found to be the most favorable one for NO, as shown in Figure 1e. The corresponding adsorption energy is determined to be -211 meV. The CO molecule somewhat favors both H and B sites, giving an identical absorption energy of -128 meV (see Figure 1g). For simplicity, the configuration at the H site is chosen as the representative for CO. All of the following results for these adsorbates are obtained based on their most favorable configurations if not specified.

**Table 1:** **Results for gas molecules on monolayer MoS**_**2**_** calculated by LDA functional**

**Gas**	**H site**			**T**_**M**_**site**			**T**_**S**_**site**			**B site**		
	***h***	***E***_***a***_	**Δ*Q***	***h***	***E***_***a***_	**Δ*Q***	***h***	***E***_***a***_	**Δ*Q***	***h***	***E***_***a***_	**Δ*Q***
H_2_	2.62	-70	0.004	2.61	-82	0.004	3.02	-49	0.008			
O_2_	2.79	-106	0.034	2.71	-116	0.041	3.19	-64	0.020			
H_2_O	2.59	-234	0.012	2.67	-222	0.016	3.13	-110	0.009			
NH_3_	2.46	-250	-0.069	2.61	-222	-0.051	3.21	-100	-0.024			
NO	2.68	-195	0.011	2.90	-185	0.011	2.88	-152	0.039	2.83	-211	0.022
NO_2_	2.65	-276	0.100				2.71	-249	0.119	2.62	-249	0.114
CO	2.95	-128	0.020	3.22	-124	0.006	3.28	-86	0.016	3.15	-128	0.013

Equilibrium height between the center of mass of the molecule and the top S-layer of the MoS_2_ sheet (*h*, in Å), adsorption energy (*E*_*a*_, in meV), and charge transfer from MoS_2_ to the molecule (Δ*Q*, in *e*). Negative Δ*Q* means charge transfer from the molecule to MoS_2_.

Additionally, calculations of the gas adsorption are also performed using GGA functional. Different from LDA functional which overestimates the adsorption energy, GGA functional usually has a tendency to underestimate it. Upon the application of the two kinds of functionals, the upper and lower bounds for adsorption energy and other structural properties can be obtained [8]. The calculated values of equilibrium height and adsorption energy for gas molecules on MoS_2_ are listed in Table 2. Herein, two GGA functionals, PW91 and PBE, are used for the purpose of comparison. Both PW91 and PBE give a smaller adsorption energy compared to the LDA, whereas they show the molecules binding at an equilibrium height larger than that for LDA. For most molecules (with the exception of NO), it seems that PW91 gives more stable results than PBE, with their adsorption energy difference approximately between -7 and -28 meV.

**Table 2:** **Results for gas molecules on monolayer MoS**_**2**_** calculated by PW91 and PBE functionals**

**Gas**	**Site**	**LDA**		**GGA-PW91**		**GGA-PBE**	
		***h***	***E***_***a***_	***h***	***E***_***a***_	***h***	***E***_***a***_
H_2_	T_M_	2.61	-82	3.21	-4	3.07	6
O_2_	T_M_	2.71	-116	3.32	-11	3.40	-4
H_2_O	H	2.59	-234	3.17	-37	3.14	-21
NH_3_	H	2.46	-250	2.99	-44	2.91	-24
NO	B	2.83	-211	3.47	-14	3.25	-33
NO_2_	H	2.65	-276	3.33	-43	3.30	-15
CO	H	2.95	-128	3.61	-13	3.62	3

Equilibrium height between the center of mass of the molecule and the top S-layer of the MoS_2_ sheet (*h*, in Å) and adsorption energy (*E*_*a*_, in meV). Positive *E*_*a*_ indicates endothermic adsorption. The LDA-calculated results are also listed for reference.

Next, Bader analysis is performed to predict the charge transfer value. It is found that most molecules studied except NH_3_ are charge acceptors with 0.004 ∼ 0.1*e* obtained from monolayer MoS_2_, whereas NH_3_ behaves as a charge donor, providing 0.069*e* to the monolayer. The charge transfer values for O_2_ and H_2_O are in good agreement with recently reported values (approximately 0.04*e* for O_2_ and 0.01*e* for H_2_O) by Tongay et al. [33]. Note that our results are somewhat similar to the previous reports on the adsorption of gas molecules on graphene [7] and carbon nanotube [34], where the gas molecules also behave as either charge acceptors or donors. We need to point out that although different methods besides Bader analysis may give rise to different values in determining the electronic charge transfer, the direction and order of magnitude should be the same. The mechanism of the MoS_2_-FET gas sensor for NO [27] can then be understood. Before NO adsorption, the mechanically cleaved MoS_2_ channel is an n-type semiconductor in the experiment, implying that some electrons have already existed in the conduction band. After NO adsorption, electron charge is transferred to the NO molecule, inducing a *p*-doping effect on the MoS_2_ channel. As a result, the channel resistance increases and current decreases. The similar behavior, which has been previously reported for MoS_2_-FET devices in an O_2_ environment [35,36], is probably due to the adsorption of O_2_ on the MoS_2_ surface, which traps electrons and sequentially reduces the current of the MoS_2_-FET.

**Figure 2:**
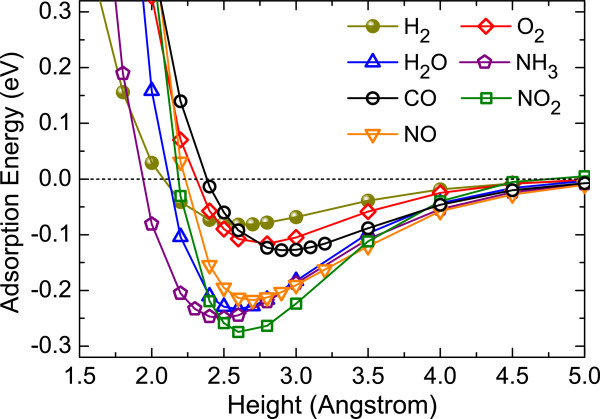
**Adsorption energy versus height.** Adsorption energy versus height between the center of mass of the molecule and the top S-layer of monolayer MoS_2_ for all the studied molecular adsorbates.

To further gain insight into the molecule-monolayer interaction, we calculate the adsorption energy curves for all the studied gas molecules, wherein the height between the center of mass of the molecule and the top S-layer of the MoS_2_ sheet is varied between 1.5 and 5.0 Å. The corresponding results are given in Figure 2. It is shown that the curve for NO_2_ gives the largest adsorption energy at the minimum, which is three times higher than that of the H_2_ curve. At equilibrium, NH_3_ has a minimum height of 2.46 Å with respect to monolayer MoS_2_, whereas CO has a maximum molecule-monolayer height of 2.95 Å. All the curves nearly reach the asymptotic value at 5.0 Å. Due to the small adsorption energy and large separation height, the interaction between the gas molecules and the MoS_2_ surface can thus be characterized as physisorption.

**Figure 3:**
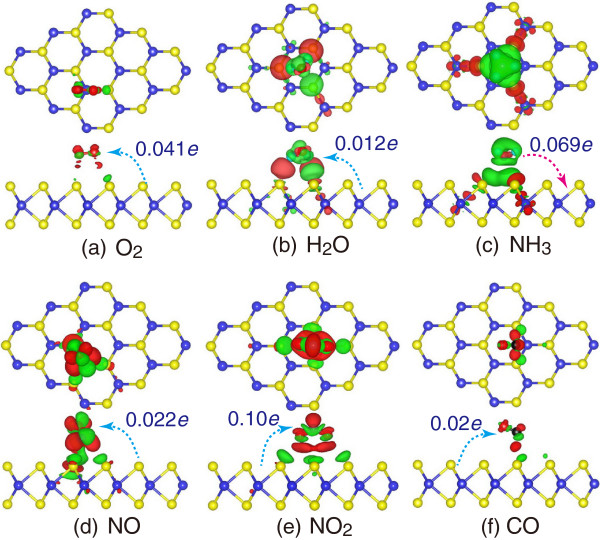
**Charge density difference plots.** Charge density difference plots for **(a)** O_2_, **(b)** H_2_O, **(c)** NH_3_, **(d)** NO, **(e)** NO_2_, and **(f)** CO interacting with monolayer MoS_2_. The red (green) distribution corresponds to charge accumulation (depletion). The isosurface is taken as 5 × 10^-4^*e*/Å^3^. The direction and value of charge transfer are also denoted.

Figure 3 presents the charge density difference images for these molecule-monolayer systems, calculated by the formula $\Delta \rho =\rho_{{\text{MoS}}_{2}+\text{molecule}}-(\rho_{{\text{MoS}}_{2}}+\rho_{\text{molecule}})$, where $\rho_{{\text{MoS}}_{2}+\text{molecule}}$, $\rho_{{\text{MoS}}_{2}}$, and *ρ*_molecule_ are the charge density of the molecule-adsorbed MoS_2_, pristine MoS_2_, and isolated molecule, respectively. The red region shows the charge accumulation, while the green region represents the charge depletion. It is shown that the MoS_2_ sheet is considerably polarized upon the adsorption of gas molecules, and electrostatic interaction plays a role in the attractive interaction. The polarization in the H_2_O, NH_3_, NO, and NO_2_ cases are stronger than that in the O_2_ and CO cases, giving rise to a larger interaction energy. It explains why the former gives larger adsorption energies (-234, -250, -211, and -276 meV for H_2_O, NH_3_, NO, and NO_2_, respectively) than the latter (-116 and -128 meV for O_2_ and CO, respectively) mentioned above.

**Figure 4:**
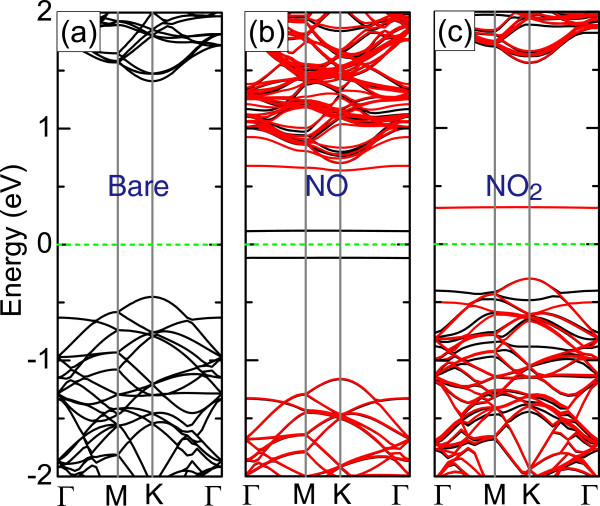
**Band structures.** Band structures of **(a)** pristine, **(b)** NO-adsorbed, and **(c)** NO_2_-adsorbed monolayer MoS_2_. The black (red) line corresponds to the up-spin (down-spin) bands, whereas the dashed green line denotes the Fermi level.

We examine the electronic properties of monolayer MoS_2_ adsorbed with gas molecules. The band structure before adsorption is presented in Figure 4a. It is found that the pristine monolayer MoS_2_ is a semiconductor with a direct band gap of 1.86 eV at K point, which is in good agreement with reported works [37-39]. The band structures for both valence bands and conduction bands of monolayer MoS_2_ are not significantly altered when H_2_O, NH_3_, and CO are adsorbed, and the gap values remain around 1.86 eV (not shown here). The situation is similar in the cases of O_2_, NO, and NO_2_ except the flat impurity states in the gap of the host monolayer induced by these adsorbates. While O_2_ introduces two close-lying down-spin states 0.519 and 0.526 eV above the Fermi level (E_F_) in the band gap, NO_2_ introduces an unoccupied down-spin state 0.31 eV above E_F_, as given in Figure 4c. Three impurity states emerge inside the band gap upon the adsorption of NO, namely, one occupied up-spin state 0.12 eV below E_F_, one unoccupied up-spin state 0.11 eV above E_F_, and one unoccupied down-spin state close to the conduction band edge with an energy separation of 0.064 eV between them (see Figure 4b). The adsorption of O_2_, NO, and NO_2_ on the MoS_2_ surface, on the other hand, creates magnetic moments of 2.0, 1.0, and 1.0 *μ*_B_ per supercell, respectively.

**Figure 5:**
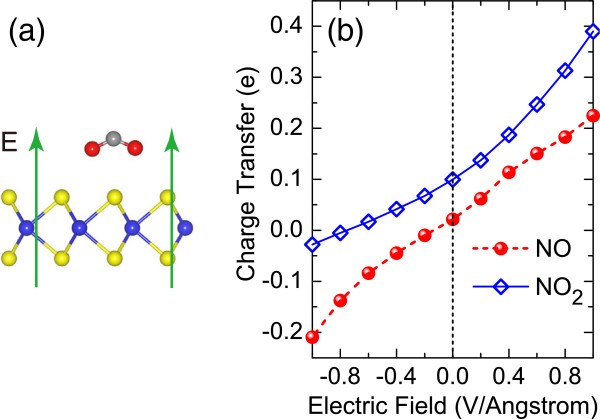
**Electric field effect.** **(a)** Representation of the applied perpendicular electric field, where the arrows denote its positive direction. **(b)** Variation of charge transfer as a function of electric field strength for NO, and NO_2_, adsorbed on monolayer MoS_2_.

As the charge transfer between the adsorbed molecule and monolayer MoS_2_ plays a crucial role in determining the performance of the MoS_2_ sensor, it may be sensitive to the applied electric field, similar to the case of graphene [40]. For brevity, NO and NO_2_ adsorbed monolayers are chosen as the representative systems. Figure 5a gives the schematic illustration of the electric field applied in our study, which is perpendicular to the plane of monolayer MoS_2_ with its positive direction aligned upward. The variation of charge transfer with respect to the electric field is shown in Figure 5b. It is found that the charge transfer from the monolayer to the adsorbed molecule increases with the increment of field strength along a positive direction, whereas it tends to decrease once reverse electric field is applied. This negative electric field will drive the electrons from the molecule to the monolayer when its field strength is beyond a critical value. While NO and NO_2_ attain 0.022*e* and 0.1*e* in the absence of electric field (*E* = 0 V/Å), respectively, they turn out to accept 0.225*e* and 0.39*e* from monolayer MoS_2_ at *E* = 1 V/Å and conversely donate 0.21*e* and 0.028*e* at *E* = -1 V/Å. The dependence of charge transfer on field direction is probably attributed to the dipole moment of the molecule-monolayer system [41]. Band structure calculations for the two systems, on the other hand, show that the impurity states in the band gap shift towards the valence or conduction bands of monolayer MoS_2_, depending on the direction of the applied perpendicular electric field.

## Conclusions

In this work, we present a first-principles study on the structural and electronic properties of monolayer MoS_2_ upon adsorption of gas molecules. Various adsorption sites and molecule orientations are involved to determine the most stable configurations. We find that all molecules are physisorbed on monolayer MoS_2_ with small charge transfer, acting as either charge acceptors or donors. Band structure calculations indicate that the valence and conduction bands of monolayer MoS_2_ is not significantly altered upon the molecule adsorption, though certain molecules such as O_2_, NO, and NO_2_ introduce adsorbate states in the band gap of the host monolayer. In addition, we demonstrate that the application of a perpendicular electric field can consistently modify the charge transfer between the adsorbed molecule and the MoS_2_ substrate. Our theoretical findings show that MoS_2_ holds great promise for fabricating gas sensors.

## Competing interests

The authors declare that they have no competing interests.

## Authors’ contributions

QY performed the first-principles calculations and drafted the manuscript. ZS and SC participated in the calculation part. JL conceived of the study and helped in writing of the manuscript. All authors read and approved the final manuscript.

## Supplementary Material

Additional file 1 **Supporting information.** Figure 1S - Possible adsorption configurations for NO adsorbed on MoS_2_. Figure 2S - Possible adsorption configurations for NO_2_ adsorbed on MoS_2_. Figure 3S - Possible adsorption configurations for NH_3_ adsorbed on MoS_2_. Click here for file
