# Supplementary material for: Adsorption of gas molecules on monolayer MoS2 and effect of applied electric field
Source: Nanoscale Res Lett. 2013 Oct 17;8(1):425. doi: 10.1186/1556-276X-8-425 (PMC4015638; doi:10.1186/1556-276X-8-425)
Supplement: Additional file 1 — Supporting information. Figure 1S - Possible adsorption configurations for NO adsorbed on MoS2. Figure 2S - Possible adsorption configurations for NO2 adsorbed on MoS2. Figure 3S - Possible adsorption configurations for NH3 adsorbed on MoS2. [file 1556-276X-8-425-S1.pdf]

Supporting information

In the calculations, a larger number of adsorption configurations have been examined to determine the more stable ones for these gas molecules adsorbed on monolayer MoS_2. For each molecule, four adsorption sites denoted as H, T_M, T_S and B are considered. For each sites, different orientations of the molecules are also involved. Herein, since CO, O_2 and H_2 have similar molecular orientations as NO, and H_2O has similar orientations as NO_2, only those adsorption configurations for NO, NO_2 and NH_3 molecules are presented for brevity [see **Figures 1S-3S**]. In the case of NO molecule, three initial molecular orientations are included: one with N-O bond parallel to the monolayer and two with N-O bond perpendicular to the monolayer, with O atom above N atom and O atom below N atom. In the case of NO_2, three different molecular orientations are included either: starting from the N atom, one with N-O bonds parallel to the monolayer, two with NO-bonds pointing up or pointing down to the monolayer. For NH_3, two orientations are considered: one with the H atoms pointing away from the monolayer and the other with the H atoms pointing to it.

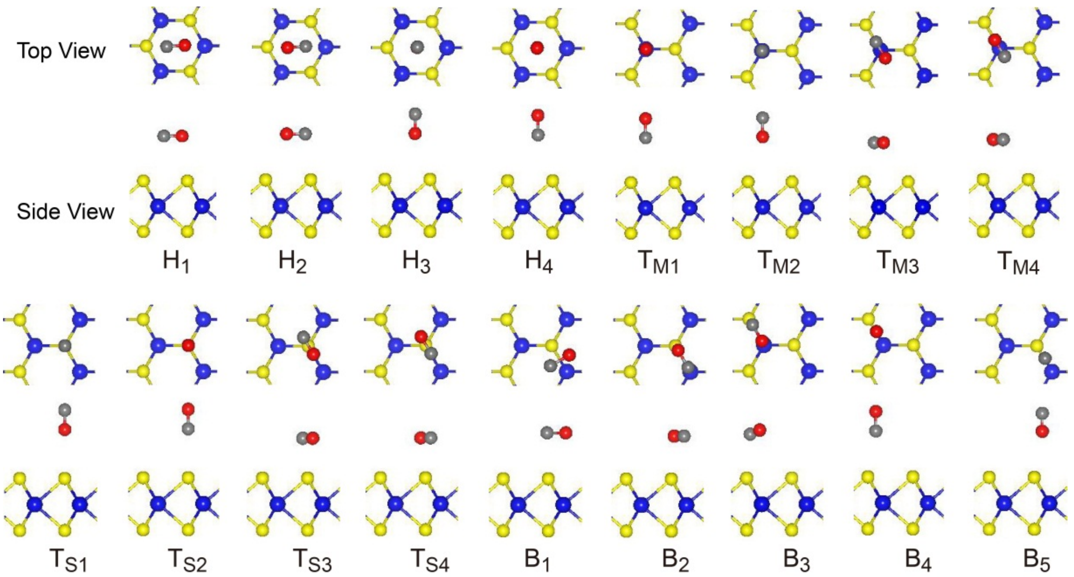

Figure 1S - Possible adsorption configurations for NO adsorbed on MoS_2. Both the top and side views are shown. The blue and yellow balls represent Mo and S atoms, while red and grey balls represent O and N atoms, respectively. H_1-H_4, T_{M1}-T_{M4}, T_{S1}-T_{S4} and B_1-B_5 denote those configurations wherein NO are absorbed at H, T_M, T_S and B adsorption sites, respectively.

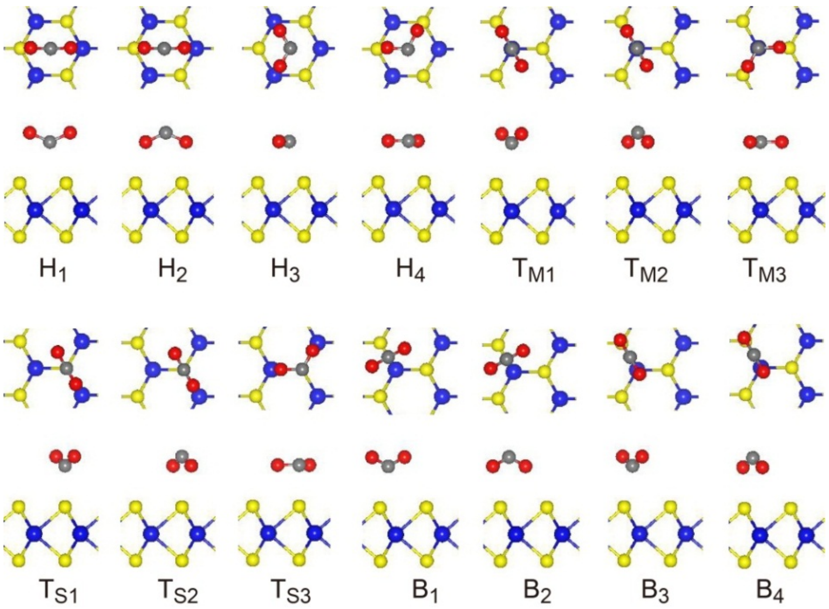

Figure 2S - Possible adsorption configurations for NO_2 adsorbed on MoS_2. Both the top and side views are shown. The blue and yellow balls represent Mo and S atoms, while red and grey balls represent O and N atoms, respectively. H_1-H_4, T_{M1}-T_{M3}, T_{S1}-T_{S3} and B_1-B_4 denote those configurations wherein NO_2 are absorbed at H, T_M, T_S and B adsorption sites, respectively.

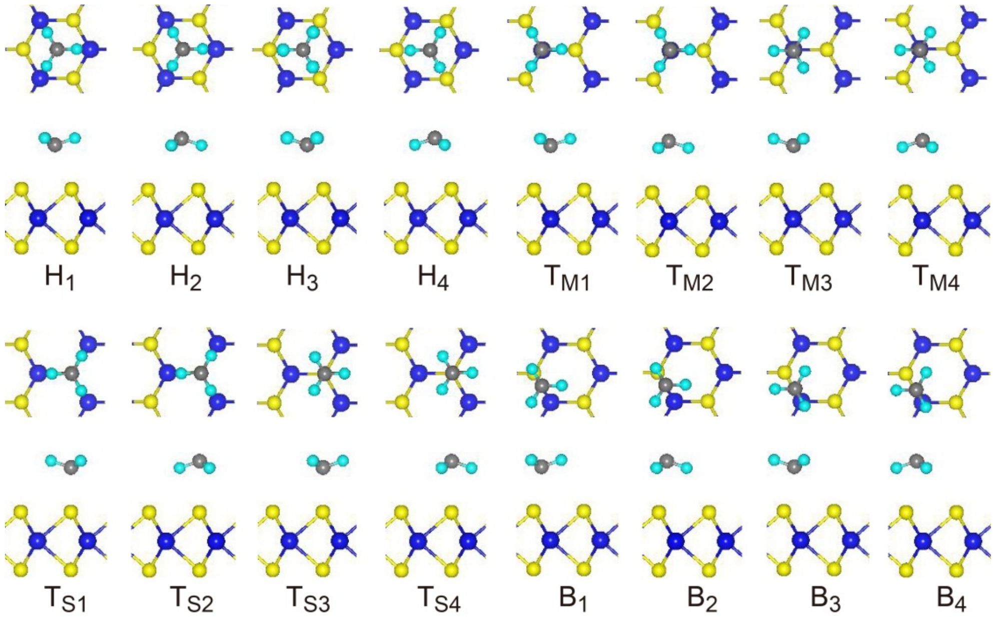

Figure 3S - Possible adsorption configurations for NH_3 adsorbed on MoS_2. Both the top and side views are shown. The blue and yellow balls represent Mo and S atoms, while cyanine and grey balls represent H and N atoms, respectively. H_1-H_4, T_{M1}-T_{M4}, T_{S1}-T_{S4} and B_1-B_4 denote those configurations wherein NH_3 are absorbed at H, T_M, T_S and B adsorption sites, respectively.
